# Multiple Connection Pattern Combination From Single-Mode Data for Mild Cognitive Impairment Identification

**DOI:** 10.3389/fcell.2021.782727

**Published:** 2021-11-22

**Authors:** Weikai Li, Xiaowen Xu, Zhengxia Wang, Liling Peng, Peijun Wang, Xin Gao

**Affiliations:** ^1^ School of Information Science and Engineering, Chongqing Jiaotong University, Chongqing, China; ^2^ Shanghai Universal Medical Imaging Diagnostic Center, Shanghai, China; ^3^ Department of Medical Imaging, Tongji Hospital, Shanghai, China; ^4^ Tongji University School of Medicine, Tongji University, Shanghai, China; ^5^ School of Computer Science and Cyberspace Security, Hainan University, Hainan, China

**Keywords:** functional connectivity, effective connectivity, multiview, multimodal, mild cognitive impairment

## Abstract

Mild cognitive impairment (MCI) is generally considered to be a key indicator for predicting the early progression of Alzheimer’s disease (AD). Currently, the brain connection (BC) estimated by fMRI data has been validated to be an effective diagnostic biomarker for MCI. Existing studies mainly focused on the single connection pattern for the neuro-disease diagnosis. Thus, such approaches are commonly insufficient to reveal the underlying changes between groups of MCI patients and normal controls (NCs), thereby limiting their performance. In this context, the information associated with multiple patterns (e.g., functional connectivity or effective connectivity) from single-mode data are considered for the MCI diagnosis. In this paper, we provide a novel multiple connection pattern combination (MCPC) approach to combine different patterns based on the kernel combination trick to identify MCI from NCs. In particular, sixty-three MCI cases and sixty-four NC cases from the ADNI dataset are conducted for the validation of the proposed MCPC method. The proposed method achieves 87.40% classification accuracy and significantly outperforms methods that use a single pattern.

## Introduction

As the most concerning neurodegenerative disease, Alzheimer’s disease (AD) comes to be the most common causes of dementia (Gaugler et al., 2016). In particular, AD can seriously interfere with patient’s daily lives, and eventually lead to deaths. Thus, a natural ambition is to delay the progression of AD during its early stages via pharmacological and behavioural interventions. In particular, mild cognitive impairment (MCI) is often considered an early indicator of potential progression to AD (Wee et al., 2012). Nearly 10–15% of patients with MCI progress to AD per year (Misra et al., 2009). Therefore, the accurate diagnosis of MCI has attracted considerable attention.

Recently, functional magnetic resonance imaging (fMRI) comes to a popular technique to reveal brain activities and patterns for the MCI diagnosis (Kevin et al., 2008). However, due to the random and asynchronous spontaneous brain activity between the subject and the scanner, it is still a challenge to identify MCI patients and normal controls (NC) based on fMRI alone. In contrast, the connectome-based methods provide a new stable biomarker which potentially helps us to understand brain information (Stam, 2014). Specifically, several studies have illustrated that several neurological diseases, such as AD (Chen et al., 2016), MCI (Gao et al., 2020), autism spectrum disorder (Li et al., 2017), and Parkinson’s disease (Abós et al., 2017) are highly related to the functional brain connections.

Notably, the exiting works are highly dependent on the estimated networks or connections. Thus, several efforts have been devoted to estimating the ideal network by incorporating additional biological priors into BCs to improve the discriminative ability of the networks, e.g., sparsity (Lee et al., 2011), scale-free priors (Li et al., 2017), modularity (Qiao et al., 2016; Li et al., 2020c; Li et al., 2020a), and group sparsity (Liang et al., 2018; Zhang et al., 2019). Moreover, the data noisy prior (Li et al., 2019) and domain knowledge prior (Li et al., 2020d) can also be adopted. However, these approaches may still be insufficient to identify MCI from NCs, since they focus only on a single connection pattern, which fails in combining the information from the multiple connections for neurological disorder diagnosis.

In this paper, we provide a simple yet valuable approach, i.e., multiple connection pattern combination (MCPC), which combines the information from multiple connection patterns to achieve a better diagnostic performance of neurological disorders. In particular, a multi-kernel support vector machine (MK-SVM) trick is employed as a naive attempt to combine the multiple connection patterns for the MCI diagnosis. Further, an MCI identification task is explored to verify the performance of the proposed MCPC method. The highlights of this paper are as follows.

1) To our best knowledge, MCPC is the first attempt that combines the multiple connection patterns to identify MCIs from NCs. The experimental results also confirm that the proposed MCPC scheme significantly outperforms single-pattern methods.
2) We identify hubs and consensus connections based on the proposed multiple connection patterns. Analyses of graph theory attributes and critical functional connectivity are performed to discriminate individuals with MCI from NCs and identify the pathological mechanism of MCI.

## Materials and Methods

### Data Preparation

The publicly available neuroimaging data from the Alzheimer’s disease Neuroimaging Initiative (ADNI)^1^ database (Jack et al., 2010) is adopted. Notably, 127 participants, including sixty-three MCIsand 64 NCs were included in this experiment. The SPM8 toolbox^2^ is used to pre-process the fMRI data according to a commonly adopted pipeline for fMRI. Finally, the pre-processed BOLD time series signals were partitioned into 116 ROIs, based on the Automated Anatomical Labeling (AAL) atlas.

### Construction of Multiple Brain Connection

We adopted the commonly-used BC estimation model to discover the connection patterns, including Pearson’s correlation (PC), sparse representation (SR) and Granger causality mapping (GCM). Let $X\in R^{T\times N}$ the BOLD signal matrix, where T is the volume length and N is the ROI number. Denote $x_{i}\in R^{T}$ the fMRI time series derived from the *i*th ROI  i=1,⋯,N . Then, the details of these methods are given as follows.

### Pearson’s Correlation

Pearson’s correlation (PC) is among the most simplicity and intuitiveness scheme for the BC estimation. The edge weights of the PC-based BC $W=\left(W_{ij}\right)\in R^{N\times N}$ is in the following:

$$W_{ij}=\frac{{\left(x_{i}-{\bar{x}}_{i}\right)}^{T}\left(x_{j}-{\bar{x}}_{j}\right)}{\sqrt{{\left(x_{i}-{\bar{x}}_{i}\right)}^{T}\left(x_{i}-{\bar{x}}_{i}\right)}\sqrt{{\left(x_{j}-{\bar{x}}_{j}\right)}^{T}\left(x_{j}-{\bar{x}}_{j}\right)}},$$ (1)

where $x_{i}-{\bar{x}}_{i}$ is a centralized counterpart of $x_{i}$ .

### Partial Correlation With Sparse Representation

Due to the cofounding effect caused by the PC-based method, the partial correlation method involves regressing complex factors from other ROIs that naturally come into being (Huang et al., 2010). Inspired by the sparsity nature of the brain connection, one popular solution is to incorporate an additional $l_{1}$ -norm constraint, resulting in a sparse representation (SR)-based BC estimation scheme, as follows.

$${min}_{W}\sum_{i=1}^{n}\|x_{i}-\sum_{j\neq i}W_{ij}x_{j}{\|}^{2}+\lambda \sum_{j\neq i}\left|W_{ij}\right|$$ (2)

where λ is the hyper-parameter for controlling the balance of sparsity and partial correlation.

### Granger Causality Mapping

Granger causality mapping (GCM) models the effective connectivity, i.e., causality relations among nodes, which connection is thereby nonsymmetric (Goebel et al., 2003). Specifically, given two-time x[n]  and y[n ], the Granger causality mapping process from x[n] to y[n] ] is defined as follows:

$$F_{x,y}=\ln\frac{\left|\sum \left(\zeta_{t}\right)\right|}{\left|\sum \left(\eta_{t}\right)\right|}\;$$ (3)

where $\zeta_{t}$ and $\eta_{t}\;$ are the residuals of the restricted and unrestricted regression models, respectively, and Σ indicates the variance.

### Combination of Multiple Connection Patterns

**FIGURE 1:**
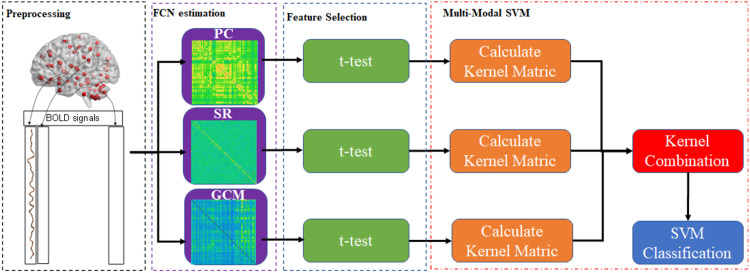
The entire framework of the proposed method for combining multiple connection patterns.

The simplest way to combine the information for multiple connection patterns is to concatenate all of the data directly. However, this approach is quite inappropriate in cases with high-dimension curves and small samples. To achieve this, this paper provided Multiple Connection Pattern Combination (MCPC), which is given in Figure 1. Specifically, an MK-SVM model is adopted to combine multiple information. Notably, this is the first attempt, which combines the information from different connectomes derived from single-mode data. Here, the primal problem of MK-SVM is given as follows: (Rakotomamonjy et al., 2007)

$$\begin{array}{l} \underset{W}{\min}\frac{1}{2}\sum_{m=1}^{M}\beta_{m}{\left\|w^{m}\right\|}^{2}+C\sum_{i=1}^{n}\xi_{i}\\ s.t.\;y_{i}\left(\sum_{m=1}^{2}\beta_{m}{\left(w^{m}\right)}^{T}\varphi^{m}\left(x_{i}^{m}\right)+b\right)\geq 1-\xi_{i}\\ \xi_{i}\geq 0,i=1\cdots ,n \end{array}$$ (4)

where n is the number of training samples and M is the number of connection patterns, $y_{i}\in \left\{1,-1\right\}\;$ representing the label of the patients or healthy controls from the *i*th sample. $\varphi^{m}$ represents the mapping function $,\;w^{m}$ represents t the hyperplane in the Represent Hilbert Kernel Space (RHKS) and $\beta_{m}$ denotes the combined weight of the *m*th connection pattern. Then, the dual form of the MK-SVM can be expressed as:

$$\begin{array}{l} \underset{\alpha }{\max}\sum_{i=1}^{n}\alpha_{i}-\frac{1}{2}\sum_{i,j}\alpha_{i}\alpha_{j}y_{i}y_{j}\sum_{m=1}^{M}\beta_{m}k^{m}\left(x_{i}^{m},x_{j}^{m}\right)\\ s.t.\sum_{i=1}^{n}\alpha_{i}y_{i}=0\\ 0\leq \alpha_{i}\leq C,i=1,\cdots ,n \end{array}$$ (5)

where $k^{m}\left(x_{i}^{m},x_{j}^{m}\right)=\varphi^{m}{\left(x_{i}^{m}\right)}^{T}\varphi^{m}\left(x_{j}^{m}\right)$ $\beta_{m}$ is learned based on Alain’s method (Rakotomamonjy et al., 2007). Additionally, we utilized the commonly-used linear kernel as a naive attempt due to its simplicity. The predictive level based on the MK-SVM can be formulated as follows:

$$f\left(x_{1},x_{2},\ldots ,x_{M}\right)=sign\left(\sum_{i=1}^{n}y_{i}\alpha_{i}\sum_{m=1}^{M}\beta_{m}k^{m}\left(x_{i}^{m},x^{m}\right)+b\right)$$ (6)

## Results

### Multiple Brain Connection Matrix Estimation From Single-Mode Data

**FIGURE 2:**
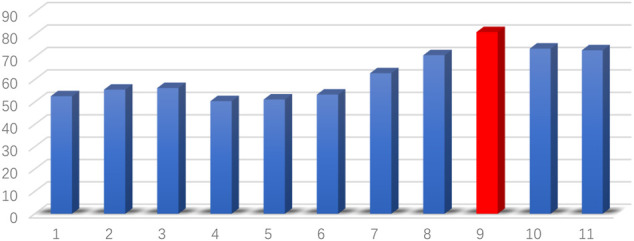
The accuracy of different hyperparameters λ for the SR.

The PC-based and SR-based BC is estimated by BrainNetClass (Zhou et al., 2020). Note that there exists a hyperparameter λ in SR. To construct the SR-based BC, we selected the hyperparameter λ the SR by leave-one-out cross-validation (LOOCV) at the range of $\left\{2^{-5},2^{-4},\ldots ,2^{5}\right\}$ . Specifically, we empirically set $\lambda =2^{3}$ , with an accuracy of 81.10%. The accuracies of different hyperparameters by LOOCV are given in Figure 2. For the GCM estimation, the dynamicBC toolbox is selected (Liao et al., 2014).

**FIGURE 3:**
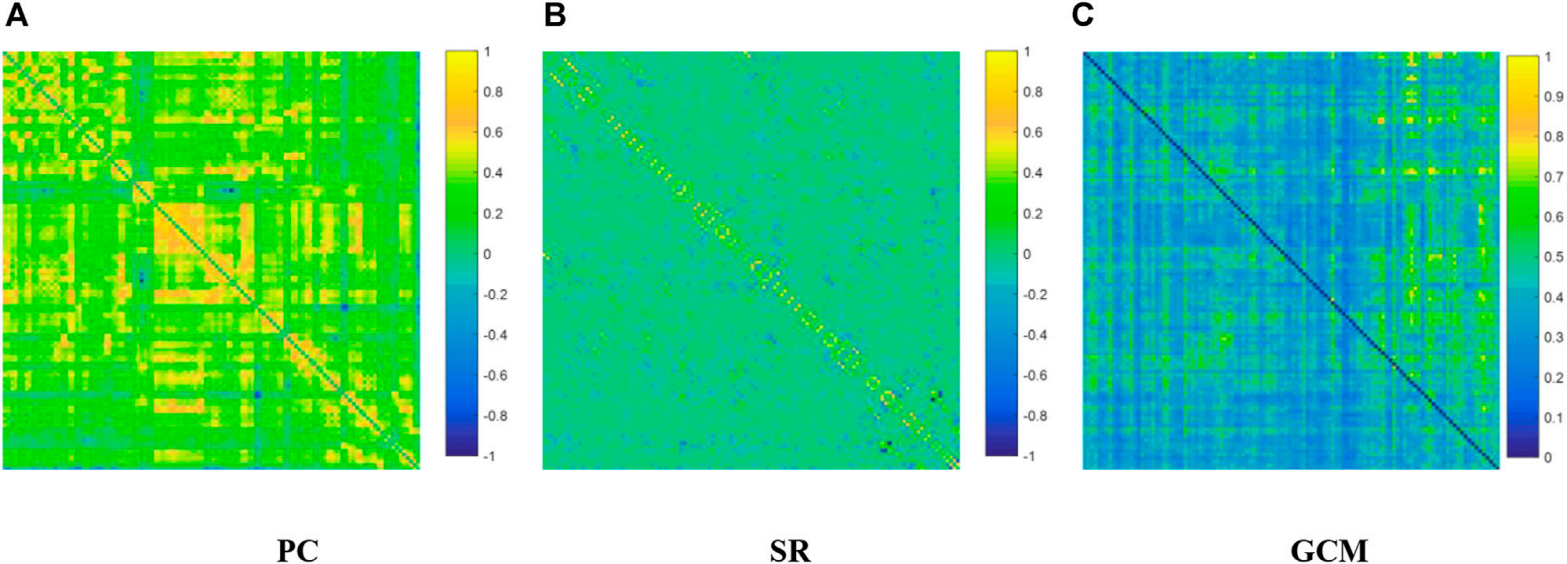
The connection networks obtained by the **(A)** PC, **(B)** SR and **(C)** GCM methods.

We visualized the BC adjacency matrices^3^ of PC, SR and GCM methods in Figure 3. In Figure 3, the brain connections obtained by different BC estimation methods are completely different in their topology, since these methods model different statistical information or relation across ROIs.

### Classification

Due to the limited sample size, we adopt the nest LOOCV strategy for evaluating the performance of the MCI classification. Specifically, to determine the optimal parameters (i.e., the optimal value of the hyperparameter C in the SVM), an inner LOOCV is conducted. The hyperparameter C is ranged in { $2^{-5},2^{-4},\ldots ,2^{5}$ }. Moreover, the accuracy, sensitivity, specificity and AUC, are used to evaluate the classification performance of different measurements. The mathematical definitions of these measurements are as follows:

$$Accuracy=\frac{TP+TN}{TP+FP+TN+FN},$$ (7)

$$Sensitivity=\frac{TP}{TP+FN},$$ (8)

$$Specificity=\frac{TN}{TN+FP},$$ (9)

Here, TP (TruePositive) is the number of the positive subjects that are correctly classified in the ASD identification task. Similarly, TN (TrueNegative), FP (FalsePostive) and FN (FalseNegative) are the numbers of their corresponding subjects, respectively.

**FIGURE 4:**
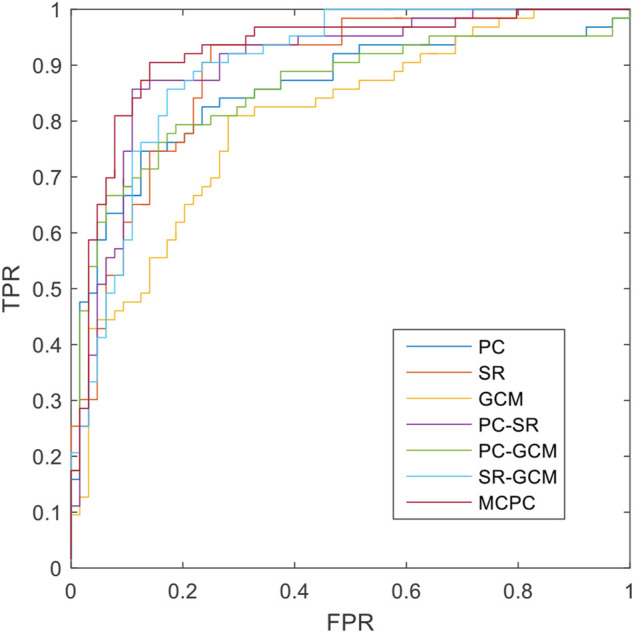
The ROCs of different methods.

The classification results based on single connection patterns are given in Table 1, which results are achieved by a single linear kernel SVM classifier. In addition, the results based on combining the partial connection patterns (e.g., PC + SR, PC + GCM and SR + GCM) are also reported. The ROC curve is given in Figure 4.

**TABLE 1:** The Classification results of different methods.

Method	Accuracy	Sensitivity	Specificity	AUC
PC	77.95	76.19	79.69	0.851
SR	81.10	84.13	78.13	0.882
GCM	72.44	66.67	78.13	0.801
PC + SR	85.83	85.71	85.94	0.898
PC + GCM	79.53	74.60	84.38	0.855
SR + GCM	82.68	80.95	84.38	0.896
MCPC	87.40	90.48	84.38	0.922

From these results in Table 1 and Figure 4, we can easily observe that the performance of MCPC achieves much better results than that of the single-kernel SVM. The results indicate the rationality of the proposed MCPC. To investigate the significance of model performance improvement, differences between various AUCs were compared by using a Delong test (Delong et al., 1988), the proposed MCPC methods are significantly superior to results of the single pattern, e.g., PC, SR, GCM under 95% confidence interval with *p*-value equals to 0.0251, 0.041 and 0.005, respectively. The superior performance illustrated that the proposed MCPC approach can significantly improve the classification performance with only single modal data. In addition, although the MCPC only use single-mode data, it can still significantly improve the accuracy of the MCI diagnosis.

### Distribution of Hubs

The hub nodes (the top 5% degree of brain nodes) of the MCI and NC groups based on three different BC network estimation methods are obtained. As shown in Tables 2-5, the distribution of hub nodes of the networks estimated by the PC, SR and GCM methods are similar. Most hubs are mainly distributed in the parietal lobes, temporal, and frontal, which correspond to the default mode network (DMN) and frontoparietal task control (FTC) network. Furthermore, the results suggest that hub nodes in the NC group are mainly located in the DMN. In comparison, the distribution of hub nodes in patients with MCI covers a relatively wide range of brain connection distributions, such as the frontoparietal task control network and visual network, in addition to the DMN.

**TABLE 2:** Hubs of the MCI and NC groups based on the PC method.

	AAL number	Corresponding brain region	Subnetwork
MCI	26	Frontal_Mid_Orb_R	DMN
	54	Occipital_Inf_R	VN
	47	Lingual_L	DMN
	24	Frontal_Sup_Medial_R	DMN
	8	Frontal_Mid_R	FTC
	9	Frontal_Mid_Orb_L	FTC
	5	Frontal_Sup_Orb_L	DMN
	22	Olfactory_R	DMN
	68	Precuneus_R	DMN
	57	Postcentral_L	SH
NC	50	Occipital_Sup_R	VN
	51	Occipital_Mid_L	VN
	48	Lingual_R	VN
	65	Angular_L	DMN
	17	Rolandic_Oper_L	CTC
	61	Parietal_Inf_L	DMN
	3	Frontal_Sup_L	DMN
	57	Postcentral_L	SH
	22	Olfactory_R	DMN
	25	Frontal_Mid_Orb_L	DMN
	34	Cingulum_Mid_R	DMN
	15	Frontal_Inf_Orb_L	DMN
	24	Frontal_Sup_Medial_R	DMN

DMN:default mode network; VN: visual network; FTC: Frontoparietal task control; SH: Sensory/somatomotor hand; CTC: Cingulo-opercular task control.

**TABLE 3:** Hubs of the MCI and NC groups based on the SR method.

	AAL number	Corresponding brain region	Subnetwork
MCI	60	Parietal_Sup_R	DAN
	18	Rolandic_Oper_R	AN
	57	Postcentral_L	SH
	8	Frontal_Mid_R	FTC
	9	Frontal_Mid_Orb_L	FTC
	20	Supp_Motor_Area_R	SH
	53	Occipital_Inf_L	VN
	2	Precentral_R	SH
NC	53	Occipital_Inf_L	VN
	50	Occipital_Sup_R	VN
	18	Rolandic_Oper_R	AN
	66	Angular_R	DMN
	4	Frontal_Sup_R	DMN
	62	Parietal_Inf_R	DMN
	12	Frontal_Inf_Oper_R	DMN
	64	SupraMarginal_R	AN

DAN: dorsal attention network; AN: auditory network.

**TABLE 4:** Hubs of the MCI group based on the GCM method.

	AAL number	Corresponding brain region	Subnetwork
MCI	62	Parietal_Inf_R	DMN
In degree	8	Frontal_Mid_R	FTC
	52	Occipital_Mid_R	DMN
	3	Frontal_Sup_L	DMN
	48	Lingual_R	VN
	29	Insula_L	SN
	37	Hippocampus_L	DMN
	49	Occipital_Sup_L	DAN
	53	Occipital_Inf_L	VN
	12	Frontal_Inf_Oper_R	FTC
	38	Hippocampus_R	DMN
Out degree	16	Frontal_Inf_Orb_R	DMN
	74	Putamen_R	SN
	85	Temporal_Mid_L	DMN
	2	Precentral_R	SH
	86	Temporal_Mid_R	DMN
	34	Cingulum_Mid_R	DMN
	55	Fusiform_L	DMN
	18	Rolandic_Oper_R	AN
	33	Cingulum_Mid_L	DMN
	41	Amygdala_L	SN
	90	Temporal_Inf_R	FTC

SN: salience network; SBN: Subcortical network; CTC: Cingulo-opercular task control.

**TABLE 5:** Hubs of the NC group based on the GCM method.

	AAL number	Corresponding brain region	Subnetwork
In degree	87	Temporal_Pole_Mid_L	DMN
	90	Temporal_Inf_R	FTC
	84	Temporal_Pole_Sup_R	DMN
	70	Paracentral_Lobule_R	SH
	14	Frontal_Inf_Tri_R	FTC
	4	Frontal_Sup_R	DMN
	23	Frontal_Sup_Medial_L	DMN
	24	Frontal_Sup_Medial_R	DMN
	41	Amygdala_L	SBN
	29	Insula_L	SN
	8	Frontal_Mid_R	FTC
Out degree	60	Parietal_Sup_R	DAN
	80	Heschl_R	AN
	79	Heschl_L	AN
	83	Temporal_Pole_Sup_L	CTC
	73	Putamen_L	SBN
	36	Cingulum_Post_R	DMN
	88	Temporal_Pole_Mid_R	DMN
	87	Temporal_Pole_Mid_L	DMN
	33	Cingulum_Mid_L	DMN
	34	Cingulum_Mid_R	DMN
	59	Parietal_Sup_L	DAN

### Consensus Connections

**FIGURE 5:**
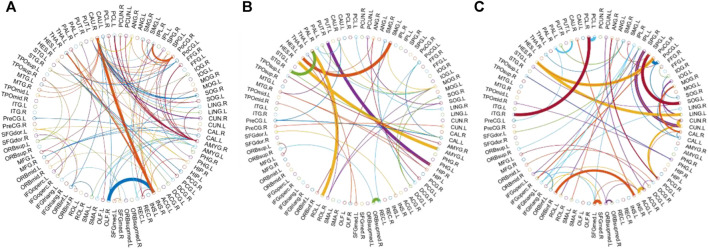
Consensus connections obtained by the **(A)** PC, **(B)** GCM and **(C)** SR methods.

In this study, the nested cross-validation scheme was adopted to evaluate the performance of the proposed MCPC. In particular, the selected connections in each validation loop might vary due to the validation resampling. Thus, we record the consensus connections and regard them as the most discriminative features for differentiating individuals with MCI from NCs (Li et al., 2020b). The consensus connections based on different connection pattern methods are shown in Figure 5. In addition, the degrees of consensus connection for different patterns are given in Tables 6-8. As shown in Tables 6-8, among the three BC estimation methods, the brain connection based on the PC method exhibits the maximum number of consensus connections. It is worth noting that the consensus connections with significant differences between MCI individuals and NCs are associated with multiple brain regions: the frontal lobe, occipital lobe, cingulate gyrus, hippocampus, and thalamus. Moreover, these brain regions corresponding to subnetworks are mainly distributed in the DMN, visual network, and subcortical network.

**TABLE 6:** Top-10 brain regions corresponding to consensus degree based on the PC methods.

AAL number	Brain region	Subnetwork	Degree
72	Caudate_R	SBN	27
42	Amygdala_R	SBN	22
30	Insula_R	SN	20
58	Postcentral_R	SH	15
80	Heschl_R	AN	12
73	Putamen_L	SBN	10
62	Parietal_Inf_R	DMN	9
15	Frontal_Inf_Orb_L	DMN	9
50	Occipital_Sup_R	VN	8
41	Amygdala_L	SBN	8

**TABLE 7:** Top-10 brain regions corresponding to consensus degree based on the SR method.

AAL number	Brain region	Subnetwork	Degree
52	Occipital_Mid_R	DMN	5
23	Frontal_Sup_Medial_L	DMN	5
58	Postcentral_R	SH	4
39	ParaHippocampal_L	DMN	4
61	Parietal_Inf_L	DMN	3
59	Parietal_Sup_L	DAN	3
35	Cingulum_Post_L	DMN	3
54	Occipital_Inf_R	VN	3
49	Occipital_Sup_L	VN	3
47	Lingual_L	DMN	3

**TABLE 8:** Top-10 brain regions corresponding to consensus connections based on the GCM method.

Direction	AAL number	Brain region	Subnetwork	Degree
In	52	Occipital_Mid_R	DMN	5
	72	Caudate_R	SBN	4
	63	SupraMarginal_L	AN	4
	77	Thalamus_L	SBN	3
	49	Occipital_Sup_L	VN	3
	43	Calcarine_L	VN	3
	38	Hippocampus_R	DMN	3
	37	Hippocampus_L	DMN	3
	78	Thalamus_R	SBN	2
	66	Angular_R	DMN	2
Out	79	Heschl_L	AN	4
	74	Putamen_R	SBN	4
	86	Temporal_Mid_R	DMN	3
	82	Temporal_Sup_R	AN	3
	52	Occipital_Mid_R	DMN	3
	33	Cingulum_Mid_L	DMN	3
	18	Rolandic_Oper_R	AN	3
	80	Heschl_R	AN	2
	76	Pallidum_R	SBN	2
	67	Precuneus_L	DMN	2

## Discussion

### Classification With Different Network Estimation Methods

From the classification results in Table 1, the SR method exhibited the highest accuracy compared to the PC and GCM methods. Although the PC method obtained more consensus connections, GCM considered more graph theory information with directions, SR achieves the best results in the single-pattern methods. These results indicated that the SR approach can effectively overcome the limitations of the PC approach. Moreover, the MCPC achieved a much better performance than the results which only utilize the single connection patterns, indicating that the proposed MCPC approach can significantly improve the diagnosis performance of MCI. Notably, different connection patterns can provide different discriminative information for diagnosis. In addition, the MCPC method outperforms the results which only considers two patterns; this result further confirms the superiority of the proposed method. Overall, as was mentioned in previous studies (Xu et al., 2020a; Xu et al., 2020b), multiple connection patterns can be combined with an MK-SVM to effectively consider the weights of different information types and differentiate MCI patients from NCs.

### The Distribution of Discriminative Features

The hub nodes of the consensus connections obtained from the three different BC estimation methods (PC, SR and GCM) are given in Tables 6-8. It can be significantly found that the most discriminative brain regions and functional connections between the MCI and NC groups were mainly distributed in the temporal, frontal and parietal lobes, which correspond to the DMN, FTC, VN, and AN. Previous studies have verified that these subnetworks correspond to various cognitive functions, such as attention, execution, and spatial positioning (Rolle et al., 2017; Bi et al., 2018). Our results suggest that patients with MCI may have altered subnetworks and corresponding cognitive functions. In particular, the DMN exhibited the most significant discriminative ability, which was consistent with previous studies of brain connections involving MCI and NC groups (Gao et al., 2020). In fact, the DMN has always been regarded as the key role for cognitive function (Anticevic et al., 2012; Liu et al., 2019). In addition, we found abnormalities in the subcortical network involving the thalamus, putamen, and amygdala in MCI. In recent years, several studies have indicated that the individuals in the early stages of AD, including subjective cognitive decline and MCI, exhibit abnormalities in subcutaneous nuclei, e.g., basal forebrain, basal ganglia, and thalamus (Fernández-Cabello et al., 2020; Xu et al., 2021). In a follow-up study, we intend to use a more detailed brain atlas than that used in this study to further explore subcortical nuclei in the early stage of AD.

## Conclusion

In this paper, we attempt to improve the performance of MCI identification by single-mode data by generating multi-view information. Specifically, we utilized the information associated with multiple brain connection patterns, which are derived from the fMRI data. The MKSVM is selected to identify the MCI from the NCs as a naive attempt, which successfully combines the information from the multiple brain connection patterns. The experimental results reveal that the MCPC strategy can significantly improve the diagnosis performance than the single pattern. Further analysis of the hub nodes and consensus connections among brain connections emphasize the importance of the DMN in the pathological mechanism associated with the early stage of AD.

## Data Availability

The original contributions presented in the study are included in the article/Supplementary Material, further inquiries can be directed to the corresponding authors.

## Footnotes

1
http://adni.loni.ucla.edu.

2
http://www.fil.ion.ucl.ac.uk.spm.

3 For the convenience of comparison among PC and SR methods, all the weights are normalized to the interval [−1, 1].
